# A Unique Case of Gallstone Ileus in a Patient With Crohn's Disease: Stone Impaction in an Ileal Adenocarcinoma

**DOI:** 10.1002/ccr3.70793

**Published:** 2025-08-11

**Authors:** Tommaso Antenucci, Rosario Arena

**Affiliations:** ^1^ Department of Medicine and Surgery University of Bologna Bologna Italy; ^2^ University Hospital Arcispedale Sant'anna of Ferrara Cona Italy

**Keywords:** Cancer, Crohn, Gallstone, Ileus, Surgery

## Abstract

Gallstone ileus represents an uncommon etiology of mechanical intestinal obstruction. Individuals with Crohn's disease are predisposed to both increased gallstone formation and a heightened risk of gastrointestinal malignancy. This case highlights the necessity of excluding malignancy when evaluating newly developed strictures in this patient population.

## 1 Introduction

Gallstone ileus (GI) is a rare mechanical bowel obstruction seen in patients with a history of cholelithiasis or cholecystitis. Although only a few cases link Crohn's disease (CD) and GI, CD increases the risk of both gallstones and gastrointestinal cancer. We present a unique case of GI resulting from stone impaction in an ileal adenocarcinoma‐related stricture in a patient with Crohn's disease.

## 2 Case History/Examination

A 61‐year‐old man with Crohn's disease presented to another hospital with nausea, vomiting, diarrhea, and low‐grade fever. He was treated for acute gastroenteritis and discharged. Two months later, he reported weight loss and bloating and was referred to our service. He had not attended follow‐up appointments since his initial diagnosis. His only medication was oral mesalamine 500 mg four times daily; his last colonoscopy was unremarkable.

## 3 Differential Diagnosis, Investigations, and Treatment

**FIGURE 1:**
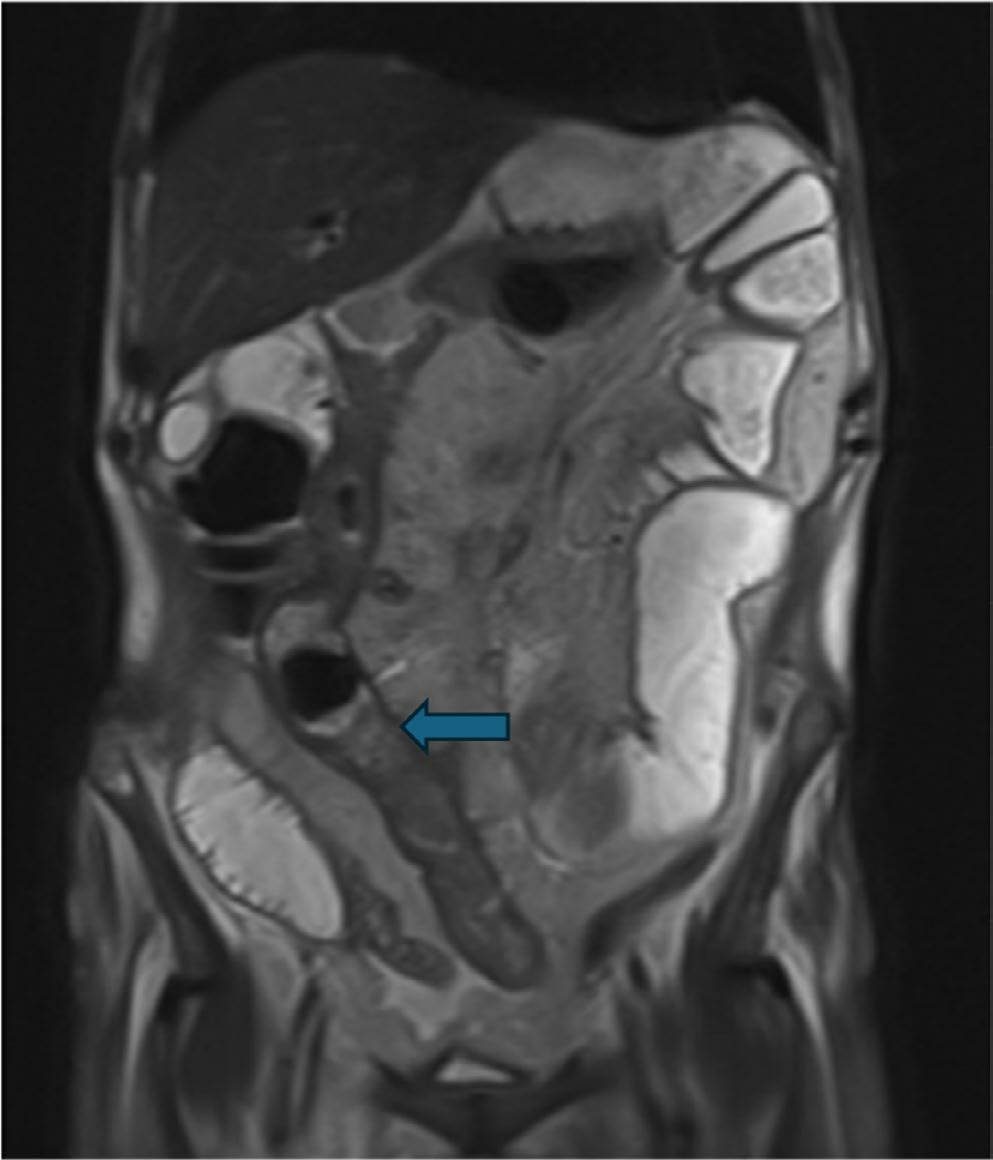
MRI image shows multiple fluid filled and enlarged loops with a hypointense formation (blue arrow) wedged in an ileal loop and causing upstream swelling.

**FIGURE 2:**
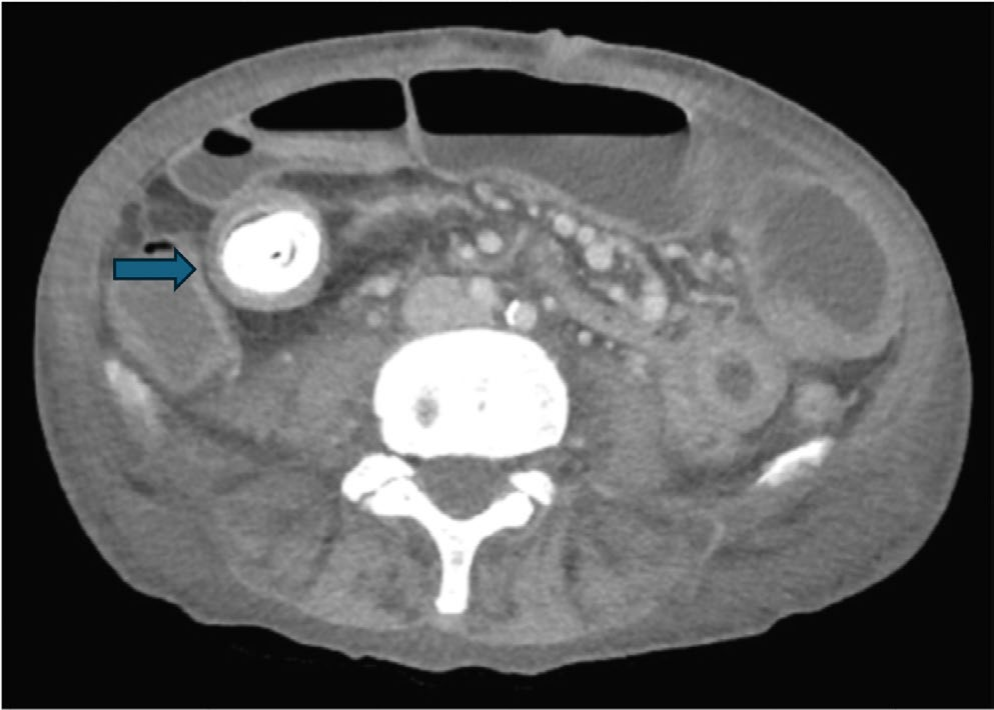
CT scan image demonstrates a stone (blue arrow) impacted in an ileal stricture.

**FIGURE 3:**
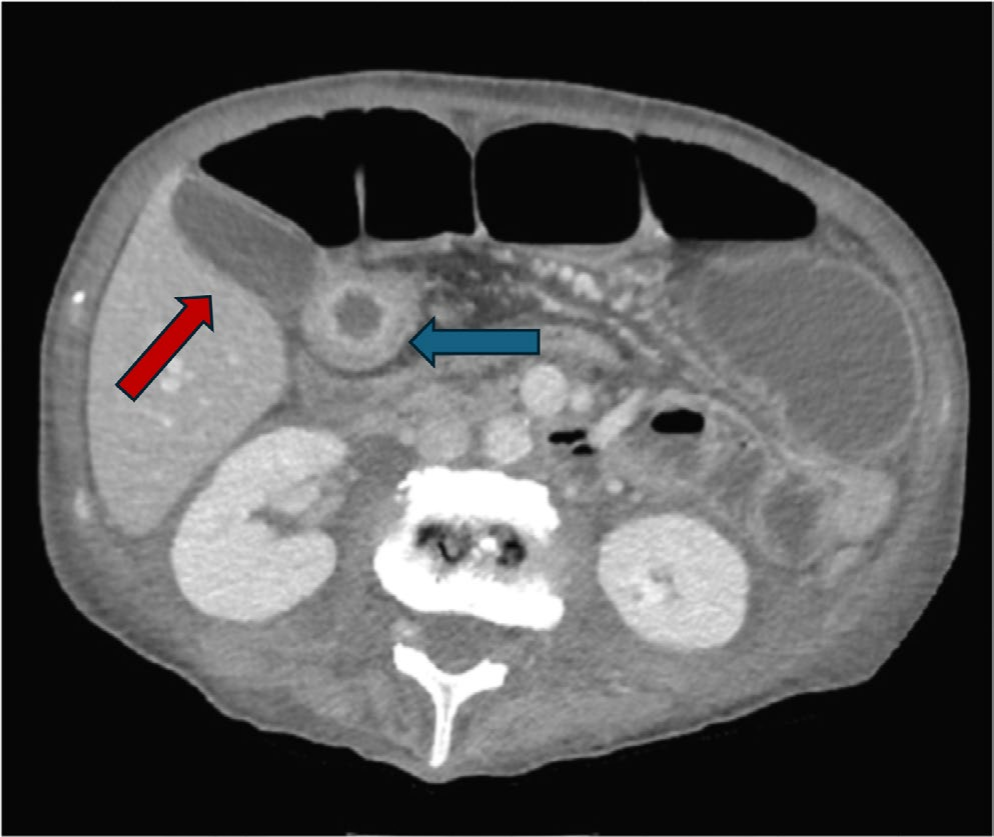
CT scan image demonstrates a thickened loop (blue arrow) in direct contact with the gallbladder wall (red arrow).

**FIGURE 4:**
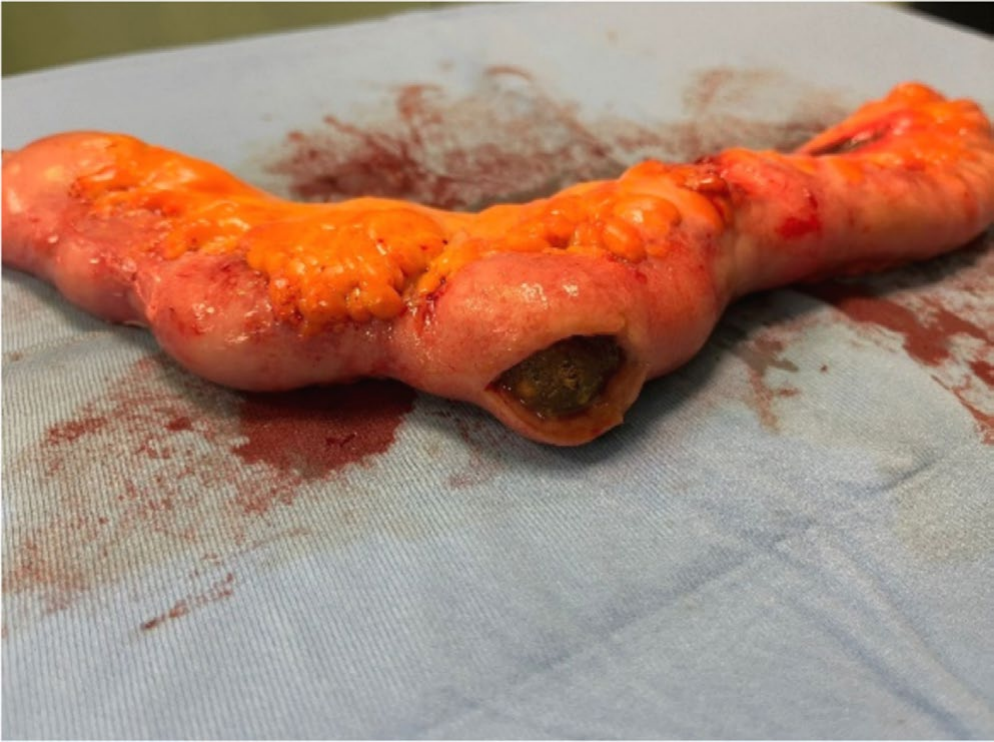
Resected ileal segment with an endoluminal stone revealed after enterotomy.

**FIGURE 5:**
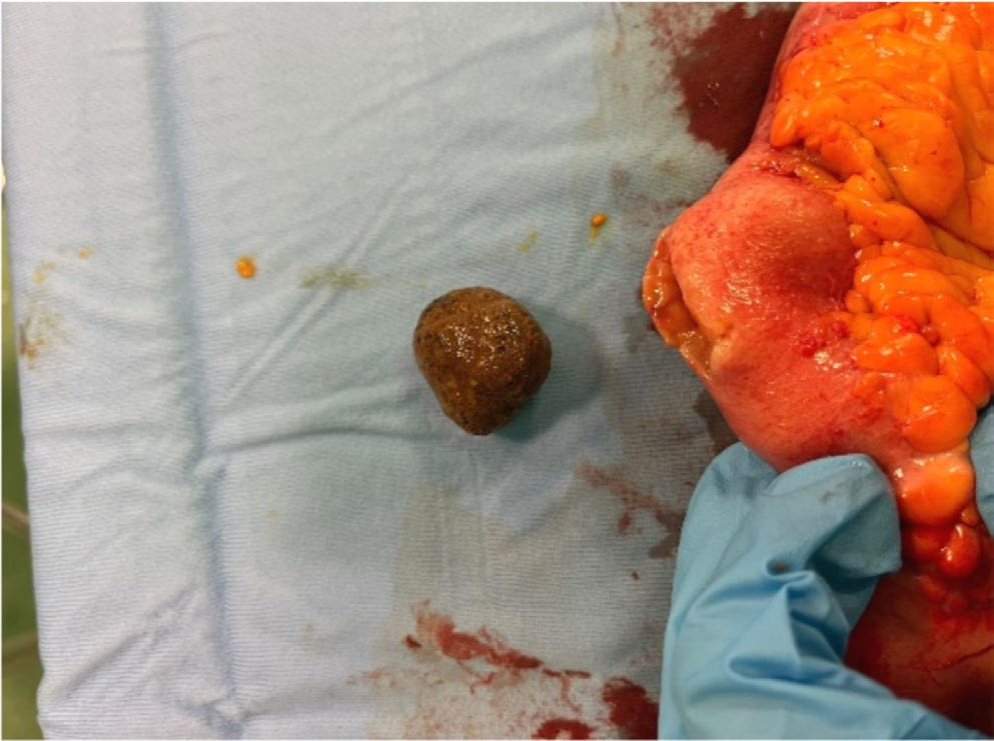
Stone after extraction.

Upon clinical evaluation, the patient was found in good overall condition, with unremarkable vital parameters. Abdominal examination found a palpable mass in the right iliac fossa with no associated signs of peritonism. Abdominal percussion revealed dullness, likely caused by fluid‐filled loops. He subsequently underwent a colonoscopy that was not completely diagnostic due to poor bowel preparation. Where visible, ileal and colonic mucosa appeared unremarkable. Biopsies taken in the ileum, cecum, ascendent, left colon, and rectum showed aspecific chronic colitis. Blood and fecal exams revealed mild microcytic anemia, elevated CPR, hypoprotidemia with hypoalbuminemia, hyponatremia, and fecal calprotectin levels > 3000 mcg/ml. Bowel MRI showed fluid distension of multiple small bowel loops located in the epigastric and mesogastric regions, with multiple hydroaerial levels and an associated thickening of a loop in the right flank stretching for about 25 cm with narrowing of the lumen (Figure 1) Ascites was present in all abdominal quadrants. After the MRI, a contrast‐enhanced CT confirmed several distended jejunal and ileal loops with a 28 × 22 mm hyperdense mass lodged in a 30 cm stenotic ileal segment (Figure 2). Above this mass, a cholecysto‐enteric fistula was seen (Figure 3), with a thickened intestinal loop in direct contact with the gallbladder. These findings were consistent with gallstone ileus. As his condition worsened, he was transferred to the emergency surgery ward where he underwent an open laparotomy in which surgeons resected a 40 cm long stenotic ileal tract, which showed initial signs of fistulization and ischemia (Figure 4). A calculus was found proximal to the stricture and extracted with an enterotomy after resection (Figure 5). Intestinal continuity restoration was achieved by the creation of an ileo‐ileal latero‐lateral anastomosis. Manual and visual exploration of the remnant bowel was unremarkable. Cholecystectomy was not performed as surgeons opted for a two‐stage approach. No adverse events occurred in the post‐operative course, and the patient was discharged.

## 4 Conclusion and Results

Histologic examination of the surgical specimen revealed a poorly differentiated adenocarcinoma infiltrating the bowel wall, the perivisceral fat, and the serosa; the lesion was present on the resection margins (R1) and metastasis was found in three lymph nodes. Phenotypic characterization showed CAM 5.2+/CDX2 + /CK20− + Cromogranin− Synaptofisin−. Final pathological staging was classified as pT4–pN2. The patient was referred to the oncological outpatient clinic where he will continue his treatment.

## 5 Discussion

The link between CD and gallstones has been established in various studies: for instance, a systematic review and meta‐analysis by Gong et al. [1] found the reported prevalence of CD patients with gallstones was approximately 14.7%. Factors significantly associated with increased gallstone risk included age ≥ 40 years (OR 3.06, 95% CI 2.09–4.48), disease duration > 15 years (OR 3.01, 95% CI 2.06–4.42), lifetime surgery (OR 2.50, 95% CI 1.99–3.12), disease located in ileocolon (OR 1.38, 95% CI 1.04–1.83) and ileocecal (OR 1.93, 95% CI 1.16–3.21), multiple hospitalizations (OR 4.26, 95% CI 2.43–7.46), corticosteroid treatments (OR 2.65, 95% CI 1.52–4.63), immunomodulator therapy (OR 1.94, 95% CI 1.12–3.38), and TPN use (OR 2.66, 95% CI 1.29–5.51). Moreover, a 330 CD consecutive case analysis by Fraquelli et al. [2] found that gallstone prevalence in CD was 24% (78 patients), with most cases involving patients who underwent resection of the ileocecal region. Another Italian study [3] found that patients with CD involving the distal ileum had the highest risk of gallstone disease (odds ratio = 4.5; 95% confidence limits = 1.5–14.1; *p* = 0.009). Proposed mechanisms of increased gallstone formation in CD include the presence of supersaturated bile with elevated levels of cholesterol, reduced cholesterol crystals and crystallization time in the gallbladder, impaired entero‐hepatic biliary acid circulation due to ileal inflammatory injury or surgical resection, and alterations of the resident microbiota [1, 4]. Small bowel carcinoma (SBC) is a very well‐documented complication of CD. In a population study by Jess et al. [5] the risk of developing SBC in CD was 60‐fold higher than in the normal population. Furthermore, a meta‐analysis from Roon et al. [6] observed that the relative risk of developing SBC in CD was 28.3, with a significant association between the anatomic location of the diseased bowel and the risk of cancer in that segment. It is known that patients with IBD are more likely to develop gastrointestinal cancers due to chronic inflammation and exposure to immunosuppressants and immunomodulators [7]. Etiopathogenesis is still unclear: proposed molecular mechanisms include mutation of the TP53 tumor suppressor gene and overexpression of p53 as a trigger for the evolution of dysplasia into neoplasia in the early stages. CD‐related SBCs also show a lower incidence of APC and KRAS mutations compared to sporadic SBCs and a higher incidence of mutations in isocitrate dehydrogenase 1 (IDH1) and SMAD4 genes. Moreover, damage to the intestinal barrier can lead to the disruption of interactions between the intestinal epithelial cells, the immune system, and the resident microbiota, thus promoting a microenvironment for tumorigenesis [8].

GI is a rare cause of mechanical intestinal obstruction and a rare complication of gallstone disease that occurs in 0.15%–1.5% of cholelithiasis cases and constitutes < 1.5% of mechanical ileus cases overall, with a recurrence rate of 5%–8% [9]. In most cases, it is caused by the formation of a cholecystoenteric fistula, which allows gallstones to translocate into the bowel, most often found in the form of a cholecystoduodenal fistula. The site of impaction can be anywhere in the GI tract, but the terminal ileum and the ileocecal valve are the most common locations because, in these locations, the lumen narrows. The treatment target is the relief of mechanical obstruction. Available strategies include surgery with enterolithotomy or segmental bowel resection, with or without concurrent biliary surgery (the so called one stage or two stage approach), endoscopic electrohydraulic or mechanical lithotripsy, and extracorporeal shockwave therapy [10, 11]. There are few reported cases of GI in CD [12, 13, 14, 15] but we could not find any reports of GI caused by a stone impaction in a narrow stricture caused by an ileal adenocarcinoma in a patient affected by CD, so our case represents a unique report. A summary of the available case reports of GI in CD is available in Table 1. All reported cases were caused by inflammatory strictures. Most of them were treated with demolitive surgery, with only one case treated with enterotomy without reconstruction. Moreover, only one case was successfully managed with conservative measures and spontaneous expulsion. We think that non‐resective procedures and conservative techniques may hinder the possibility of recognizing malignancy in a newly found stricture, so this kind of treatment should be reserved for selected cases. In fact, our case perfectly summarizes the importance of not underestimating a CD‐related stricture, as it may hide malignancy and jeopardize patient outcomes if left unrecognized.

**TABLE 1:** A resume of the currently available case reports of gallstone ileus in Crohn's disease patients.

Author	Year	Etiology	Treatment
Qureshi et al. [12]	2009	Stone impaction at ileo‐colonic junction	Conservative measures
Toelen et al. [13]	2012	Stone impaction at ileo‐colonic junction	Ileal and colonic resection
Basili et al. [14]	2006	Stone impaction in ileal stricture	Enterotomy with stone extraction
Highman et al. [15]	1981	Stone impaction at ileo‐colonic junction (two cases)	Ileal and colonic resection

## 6 Conclusion

GI is an uncommon cause of mechanical obstruction in the general population; however, individuals with CD may have a higher risk due to an increased prevalence of gallstones. This case also illustrates the need to consider malignancy as a possible cause for any new stenosis detected in CD patients, given their higher risk of gastrointestinal cancer.

## Author Contributions

**Tommaso Antenucci:** conceptualization, investigation, supervision, validation, visualization, writing – original draft, writing – review and editing. **Rosario Arena:** conceptualization, resources, supervision, validation, visualization, writing – original draft, writing – review and editing.

## Consent

Written informed consent was obtained from the patient to collect personal clinical data, write the paper, and send it for consideration and publication in a scientific journal.

## Conflicts of Interest

The authors declare no conflicts of interest.

## Data Availability

No generated or analysed data was used as this is a case report. Data cited for the discussion section is available in the original cited bibliography.
